# FEASIBILITY OF AN EVIDENCE-BASED MENTAL HEALTH INTERVENTION WITH OLDER ADULTS IN POST-HURRICANE MARIA PUERTO RICO

**DOI:** 10.1093/geroni/igz038.1993

**Published:** 2019-11-08

**Authors:** Denise Burnette

**Affiliations:** Virginia Commonwealth University, Richmond, Virginia, United States

## Abstract

Owing to out-migration, decreased fertility and longer lifespans, 18.5% of Puerto Rico’s population is aged 60+ -- 36% live alone and 40% are below poverty. Out-migration after Hurricane Maria may well raise the proportion of older adults to 30%. Mental health problems are among the most widespread and enduring effects of disasters. Common Mental Disorders (CMD) (anxiety, depression, traumatic stress) are most common. Before Maria, CDC data showed 38 % of persons age 65+ reported fair or poor health and 20% had been told they had a depressive disorder. In the months after Maria, the overall suicide rate rose 29%, while it more than doubled for people aged 65-69 and tripled for those aged 75- 79. This pilot study assesses the feasibility and acceptability of adapting the Friendship Bench, an evidence-based intervention developed in Zimbabwe for CMD, for use with older adults in primary care in Puerto Rico.

